# Conventional Meso-Scale and Time-Efficient Sub-Track-Scale Thermomechanical Model for Directed Energy Deposition

**DOI:** 10.3390/ma15124093

**Published:** 2022-06-09

**Authors:** Vaibhav Nain, Thierry Engel, Muriel Carin, Didier Boisselier

**Affiliations:** 1 Irepa Laser, Parc d’Innovation, 67400 Illkirch-Graffenstaden, France; db@irepa-laser.com; 2 Univ. Bretagne Sud, UMR CNRS 6027, IRDL, 56100 Lorient, France; muriel.carin@univ-ubs.fr; 3 Institut National des Sciences Appliquées, 67084 Strasbourg, France; thierry.engel@insa-strasbourg.fr

**Keywords:** additive manufacturing (AM), directed energy deposition (DED), distortion prediction, numerical modeling, finite element analysis (FEA), stress relaxation, COMSOL Multiphysics

## Abstract

Thermally-induced distortion and residual stresses in parts fabricated by the additive manufacturing (AM) process can lead to part rejection and failure. Still, the understanding of thermo–mechanical behavior induced due to the process physics in AM process is a complex task that depends upon process and material parameters. In this work, a 3D thermo-elasto-plastic model is proposed to predict the thermo–mechanical behavior (thermal and distortion field) in the laser-directed energy deposition (LDED) process using the finite element method (FEM). The predicted thermo–mechanical responses are compared to stainless steel 316L (SS 316L) deposition, with single and double bead 42-layer wall samples subject to different inter-layer dwell times, which govern the thermal response of deposited parts in LDED. In this work, the inter-layer dwell times used in experiments vary from 0 to 10 s. Based on past research into the LDED process, it is assumed that fusion and thermal cycle-induced annealing leads to stress relaxation in the material, and is accounted for in the model by instantaneously removing stresses beyond an inversely calibrated relaxation temperature. The model predicts that, for SS 316L, an increase in dwell time leads to a decrease in in situ and post-process distortion values. Moreover, increasing the number of beads leads to an increase in in situ and post-process distortion values. The calibrated numerical model’s predictions are accurate when compared with in situ and post-process experimental measurements. Finally, an elongated ellipsoid heat source model is proposed to speed up the simulation.

## 1. Introduction

Over the last two decades, the laser-directed energy deposition (LDED) additive manufacturing (AM) process has grown tremendously due to its possibility of fabricating large complex parts, repairing damaged components, and adding corrosion resistant coatings. However, due to the physics of the LDED process, the generation of thermal stresses and distortion in the workpiece is unavoidable. The accumulation of distortion during and after the fabrication process adversely affects the part’s dimensional accuracy, sometimes leading to its rejection. This is a critical problem that is hindering the possibility of large-parts fabrication employing the LDED process. Therefore, it is important to understand the evolution of residual stress and distortion that depends upon process parameters and material. In an effort to understand these complex phenomena, researchers have employed in situ monitoring techniques to study the effects of process parameters and analyze distortion in LDED-fabricated workpieces. Denlinger et al. [1] used a laser displacement sensor (LDS) at the free end of a cantilever substrate to measure and analyze the in situ distortion for Ti-6Al-4V and Inconel 625 deposition for different inter-layer dwell times. They concluded that longer dwell times result in an increase in distortion values for Inconel 625, and present a contrary trend for Ti-6Al-4V. Heigel et al. [2] also employed LDS at the free end of a substrate to measure and analyze the in situ distortion for Inconel 625 for different laser power, laser scan speed and deposition pattern. They concluded that distortion accumulation in LDED is more complex, as compared to a similar welding technology. With regard to measurement of in situ distortion at the deposited layers, digital image correlation (DIC) systems were used. Ocelik et al. [3] used DIC systems to analyze in situ strain field on the back side of a specimen for laser cladding. They observed that the longitudinal strain at the bottom surface of the substrate decreases with an increase in the laser scanning speed. Xie et al. [4] observed in situ full-field strain via the DIC method for the fabrication of Ti-6Al-4V walls, and discovered that the vertical strain was significantly higher than the longitudinal strain of the material just below the current deposited layer.

However, this experimental trial and error approach is time- and resource-consuming, which reduces the competitive edge of LDED. On the contrary, numerical modeling of the LDED process can be a better approach to understand and predict the thermo–mechanical behavior, as well as to fabricate parts as quickly as possible. A lot of research effort has been conducted for the development of a thermo–mechanical model for the LDED process. Denlinger et al. [5,6] validated the thermo–mechanical model for Ti-6Al-4V and Inconel 625 with experimental in situ LDS results at the substrate. They demonstrated the model effectiveness by accurately capturing the effects of inter-layer dwell times for both Inconel 625 and Ti-6Al-4V. Lu et al. [7] demonstrated a thermo–mechanical model for Ti-6Al-4V complex geometries, and validated the model with in situ LDS results at the substrate. They found that the complex geometry (S-shape) yielded an asymmetrical stress distribution. Xie et al. [8] developed and validated an experiment-based stress relaxation thermo–mechanical model for Ti-6Al-4V with experiment in situ LDS results at the substrate. They concluded that the thermo–mechanical model combined with the stress–relaxation model yielded more accurate results, as compared to a conventional thermo–mechanical model for LDED. Biegler et al. [9,10,11] validated the thermo–mechanical model for SS 316Lwith in situ DIC results at the deposited part (simple walls and curved shape). They demonstrated an approach to accurately predict distortions for industrial-scale LDED parts. Xie et al. [12] also validated the thermo–mechanical model for Ti-6Al-4V walls with in situ DIC results at the deposited wall. They found that the strain magnitude was sensitive to the location of a thin wall.

The effect of phase transformation due to solid-state phase transformation in steel, Ti-6Al-4V, and other materials leading to stress relaxation (SR) in LDED is well studied [6]. Furthermore, liquefaction of the material (feedstock) contributes to stress relaxation in LDED [9]. Finally, due to the process physics governing LDED, a fabricated component experiences multiple thermal heating and cooling cycles for a considerable amount of time, leading to annealing-induced stress relaxation, and has been extensively studied [13,14]. Hence, it is convenient to consider the stress relaxation in LDED that alters the mechanical behavior of the material during the fabrication.

Denlinger et al. [5] proposed an instantaneous stress–relaxation model for Ti-6Al-4V using electron beam DED to account for transformation strain and stress relaxation. The model works by setting both plastic strain and stress to zero if the computed temperature goes beyond the inversely calibrated prescribed relaxation temperature. Moreover, Denlinger et al. [6] demonstrated that an instantaneous stress–relaxation model for Ti-6Al-4V predicts the correct distortion accumulation, the result of which is also consistent with the experiment results for LDED technology. Some other researchers have also used the same methodology of using an instantaneous stress–relaxation model for Ti-6Al-4V in the LDED process [15,16]. However, in the LDED or AM process, the actual relaxation behavior is a time-transient temperature-dependent gradual process [17]. Xie et al. [8] developed an experiment-based stress–relaxation model for Ti-6Al-4V for use in LDED. Experiment-based methodology is coherent to the physical stress relaxation behavior of the material that is dependent on time and temperature in LDED.

Griffith et al. [18] concluded that, during the deposition of H13 tool steel in the LDED process, a sufficiently high temperature is reached to cause the material to anneal, thereby leading to stress relaxation. Kim et al. [19] experimentally and numerically investigated that, during the deposition of stainless steel 316L (SS 316L) in powder bed fusion (PBF) AM technology, there is a significant stress relaxation due to thermal cycles which are induced from a subsequent deposition of layers. Biegler et al. [9] proposed to reset the material’s plastic and stress history above solidus temperature to account for stress relaxation due to the liquefication of the SS 316L deposition in LDED.

Currently, modeling techniques (thermo–mechanical models) based on finite element method (FEM) are mostly adopted to perform thermo–mechanical analysis, with the aim of accurately predicting the thermo–mechanical response (temperature, distortion, and residual stresses) of the deposited metal during the LDED process. To date, to the best of the authors’ knowledge, few researchers have developed and validated a thermo–mechanical model based on the FEM with stress relaxation for SS 316L which presents in situ and post-process distortion experimental results in LDED technology. Currently, the use of numerical simulation for LDED is limited, due to the impractical computational times for large parts, the lack of validated thermo–mechanical material properties, and issues pertaining to the in situ measurements that are then used to calibrate and validate the numerical model.

Therefore, in the present work, the main objective is to develop a thermo–mechanical model with an instantaneous stress relaxation temperature model that also accounts for the liquefaction of the material and thermal cycle-induced stress relaxation for SS 316L in the LDED process. The prescribed stress relaxation temperature is calibrated by performing an inverse calculation, and comparing it with in situ distortion results. At first, the numerical model’s heat source parameters, boundary heat losses, and instantaneous stress relaxation temperature values were calibrated with one set of experiment results. Then, the calibrated thermo–mechanical model was verified with different experiment results, obtained through a set of experiments with varying inter-layer dwell times and numbers of beads. The thermo–mechanical model was validated with in situ temperature and distortion experiment data obtained during the fabrication of a 42-layer-high, single- and double-beaded wall on a cantilever substrate with inter-layer dwell times of 0, 5, and 10 s. The thermo–mechanical model is further validated through post-process distortion results. Furthermore, Nain et al. [20] have demonstrated that thermal modeling with an elongated ellipsoid (EE) heat source works efficiently by considerably reducing the computation time required for a pure thermal model. Therefore, in the present work studying a thermo–mechanical model, the effectiveness of this approach (the EE heat source) is also demonstrated by efficiently predicting temperature and distortion history with a drastic reduction in computation time.

## 2. LDED Modeling Approach

The proposed thermo–mechanical model focuses primarily on thermal and mechanical fields. The geometry of the different layers of the deposited wall is supposed to be known from the experiments previously conducted. The numerical model discretizes the continuous physical process of material deposition in a combination of successive simulation steps, in which laser travel is considered as a sequential step-by-step process. The numerical simulations of the LDED process are performed sequentially: firstly, a 3D transient thermal analysis is performed to obtain the temperature field, assuming a weak thermal–mechanical coupling. Then, thermal results are applied as a thermal load to a 3D quasi-static mechanical analysis to simulate the mechanical response. The thermo–mechanical model for LDED can be considered as weakly coupled (one-way coupling) due to the fact that the laser energy source in thermal analysis is much higher than the plastic strain energy in mechanical analysis [21]. On the contrary, the fully coupled (two-way coupling) thermo–mechanical model performs a transient thermo–mechanical analysis at each time step, thereby drastically increasing the computation cost.

**Figure 1:**
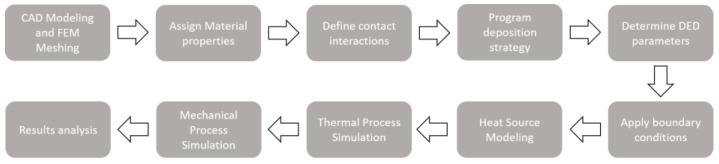
Flowchart of the proposed thermo–mechanical model.

The entire model is built on COMSOL Multiphysics 5.6 software, with the successive steps shown in Figure 1.

### 2.1. Thermal Analysis

At any point located by r in a Lagrangian domain Ω, the temperature, T, as a function of time, t, is obtained by solving the governing differential equation for transient heat transfer analysis, expressed as:

(1) $$\rho \left(T\right)C_{P}\left(T\right)\frac{\partial T\left(r,t\right)}{\partial t}=-\nabla \cdot q\left(r,t\right)+Q\left(r,t\right),\;r\in \Omega$$

where ρ(T) is the temperature-dependent material density, $C_{P}$ is the temperature-dependent specific heat capacity, Q is the volumetric heat source, and q is the heat flux vector, given as:

(2) q=−k(T)∇T

Here, k(T) represents the temperature-dependent thermal conductivity of the material which is assumed to isotropic in the model. To represent laser energy, numerical heat input is expressed by a moving volumetric double ellipsoid (DE) model [22] heat source:

(3) $$Q=\frac{6\sqrt{3}{\mathrm{APf}}_{f,r}}{a_{f,r}\mathrm{bc}\pi \sqrt{\pi }}\exp\left(-\frac{3{\left(x+v_{s}t\right)}^{2}}{a_{f,r}{}^{2}}-\frac{3y^{2}}{b^{2}}-\frac{3z^{2}}{c^{2}}\right)$$

where P is the laser power, A is the absorption efficiency that is calibrated with the experiment results, and f is a weighting fraction that governs the energy division between the front and rear ellipsoid. The parameters a, b, and c represent the melt-pool’s longitudinal, transverse, and depth dimensions of the ellipsoid, respectively. The DE model moves at $v_{s}$ velocity, and its origin is centered where the heat source reaches its maximum intensity.

To account for convective and radiative heat losses in the model, the following boundary conditions (Figure 1: depicted in flowchart) are applied on all surfaces, using Newton’s and the Stefan–Boltzmann laws:

(4) $$q_{\mathrm{loss}}=h\left(T_{s}-T_{\mathrm{amb}}\right)+\;\varepsilon \sigma \left(T_{s}^{4}-T_{\mathrm{amb}}^{4}\right)$$

where h is the convective heat transfer coefficient in (W/m^2^K), $T_{s}$ is the surface temperature, $T_{\mathrm{amb}}$ is the ambient temperature i.e., 20 °C, ε is the surface emissivity, and σ is the Stefan–Boltzmann constant.

The effect of the melt-pool flow is modeled using an enhanced thermal conductivity factor of 2.5 if the calculated temperature exceeds the fusion temperature of the material, as specified in the literature [20]. Moreover, the effect of latent heat from fusion is accounted for by modifying the specific heat, as specified in the literature [20]. It was verified that this value provided a similar melt pool length between the model and the measurement obtained by the infrared camera.

Temperature-dependent material properties for SS 316L are employed in the model, which was taken from the literature, and are presented in Table 1 [11].

### 2.2. Mechanical Analysis

The governing mechanical stress equilibrium equation can be given as:

(5) ∇·σ=0

The mechanical constitutive law where the stress–strain relationship of the material is described using Hooke’s law of linear elastic material:

(6) $$\sigma =C\varepsilon_{e}$$

where C is the fourth order elasticity tensor with a temperature-dependent Young’s Modulus, **E**, as given in Table 1, and Poisson’s ratio ν is taken as 0.3 [11]. The total strain **ε**, considering small deformation theory and thermo–elasto–plasticity, is decomposed additively in elastic $\varepsilon_{e}$ and inelastic part $\varepsilon_{in}$:

(7) $$\varepsilon =\varepsilon_{e}+\varepsilon_{in}$$

Inelastic strain includes thermal $\varepsilon_{th}$ and plastic strain $\varepsilon_{pl}$ in the numerical model.

(8) $$\varepsilon_{in}=\varepsilon_{th}+\varepsilon_{pl}$$

Thermal strain is computed using the temperature-dependent coefficient of thermal expansion α:

(9) $$\varepsilon_{th}=\alpha \;\left(T-T_{\mathrm{ref}}\right)$$

where $T_{\mathrm{ref}}$ is the reference temperature. The plastic strain is calculated by employing the von Mises yield criterion and the isotropic non-linear hardening model:

(10) $$F=\sigma_{\mathrm{vm}}-\sigma_{y}\left(\varepsilon_{\mathrm{eq}},T\right)\leq 0$$

where F is the yield function, $\sigma_{\mathrm{vm}}$ is the von Mises stress, $\sigma_{y}$ is yield stress, and $\varepsilon_{\mathrm{eq}}$ is the equivalent plastic strain.

(11) $$\sigma_{y}\left(\varepsilon_{\mathrm{eq}},T\right)=\sigma_{y0}\left(T\right)+\sigma_{\mathrm{sat}}\left(1-e^{-{\beta \varepsilon }_{\mathrm{eq}}}\right)$$

Voce’s hardening law was used to model non-linear isotropic hardening, as presented in Equation (11), where $\sigma_{y0}$ is the temperature-dependent initial yield stress, and $\sigma_{\mathrm{sat}}$ and β are saturation flow stress and saturation exponent, respectively. The values of these two parameters were extracted from stress–strain data presented in the literature [9].

In the numerical model, the effect of phase transformation is neglected, since the SS 316L remains austenitic at all temperatures [23]. However, to account for the effects of liquefication and annealing-induced stress relaxation (SR) in the SS 316L, in the numerical model, the instantaneous stress relaxation (SR) model is proposed. It is implemented by re-setting the equivalent plastic strain $\varepsilon_{\mathrm{eq}}$ to zero, thereby making it lose its hardening history, provided that the norm of the temperatures at all nodes of an element is higher than the relaxation temperature T_relax_. The stress relaxation (SR) temperature is set to 1000 °C after performing the reverse calibrations, as advised in the literature [5]. In addition, to account for liquefication’s effect in the melt-pool, the effect of thermal strain is negated by resetting the value of α to zero if the calculated temperature of an element exceeds the fusion temperature of SS 316L (1450 °C).

## 3. Experiment Set-Up

The numerical modeling approach explained in the previous section was applied to simulate the thermo–mechanical responses during the deposition of SS 316L. A detailed explanation of the experiment set-up and process parameters is provided in this section. Single- and double-adjacent beads thin wall structures of SS 316L were deposited on a substrate measuring 100 mm long, 50 mm wide, and 3 mm thick of the same material as the wall built through the LDED process. An in-house-developed machine named MAGIC was used for the LDED system, equipped with a 2 kW diode laser through the IPG laser system. To achieve the process stability, the laser and powder were co-focused on the top surface of the substrate. The laser (measured by a beam analyzer) and the incoming powder (measured by the weight measurement method) had top-hat and gaussian distributions, respectively.

**Figure 2:**
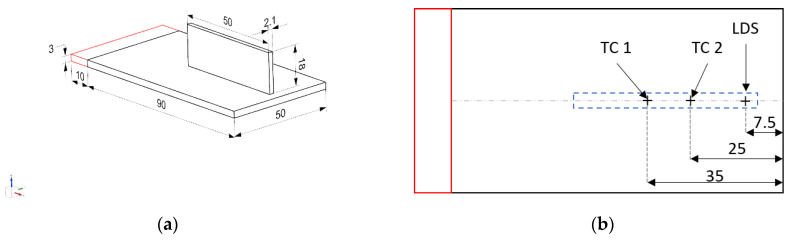

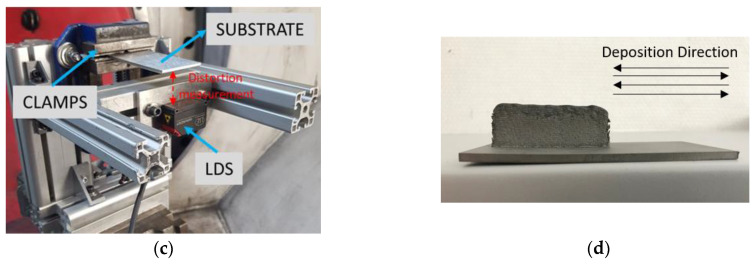
(**a**) Substrate and wall build dimensions (in mm); (**b**) Measurement locations at the substrate; (**c**) Experimental set-up of the in situ measurement; (**d**) Post-process wall build along with the depiction of the deposition strategy.

All of the experiments were performed with a scanning speed of 1 m/min and a zig-zag deposition strategy. A powder deposition rate of 13 g/min for SS 316L powder feedstock (Oerlikon, grain size 45–106 µm) was chosen. The diameter of the laser beam spot size was 2.2 mm in diameter at the top-surface of the substrate. Figure 2a shows the schematic of the substrate’s dimensions, along with clamping conditions and the planned wall build for experiment case 1. Figure 2b shows the in situ measurement locations for the thermocouple and laser displacement sensor on the bottom face of the substrate. Figure 2c shows the tooling with the substrate fixed to a clamp and a laser displacement sensor attached to the tooling. Figure 2d shows the wall build obtained for experiment case 1, along with a schematic of the deposition direction. Each wall build was 42 layers high, with a longitudinal zig-zag deposition strategy.

The effect of the waiting time (dwell time) between successive layers was analyzed, keeping laser power 800 W and laser scan speed 1 m/min fixed. Dwell times of 0, 5, and 10 s were taken between the deposition of successive layers to expose the workpiece to different cooling times. The effect of dwell time was studied on two different wall builds, both a single- and double-bead wall. Experiment cases with different process parameters are summarized in Table 2.

### 3.1. Temperature Measurement

Omega GG-K-30 type K thermocouples measuring 250 µm in diameter were employed to measure the in situ temperature. For this, two different locations were chosen at the bottom face of the substrate to record the temperature evolution, as shown in Figure 2b, so that they would fall under the deposition path of the laser on the top surface of the substrate. The thermocouples employed in the experiments had a measurement uncertainty of ±0.75%. The National Instruments module 9213 was used to read the thermocouple signals. The module recorded data at a sampling rate of 200 Hz. The recorded data were acquired through SignalExpress 2013 software, and analyzed in Igor Pro 8 software.

### 3.2. In Situ Distortion Measurement

The experimental set-up and tooling were designed to clamp the substrate from one end, and to let it distort at the free end during and after the deposition process, as shown in Figure 2c. A Micro-Epsilon 1420 laser displacement sensor (LDS) with a linear accuracy of ±8 µm was attached to the tooling to record the in situ deflection of the substrate in the build direction i.e., z-direction. The exact measurement location of the LDS sensor is shown in Figure 2b. The LDS optical signals were read and converted through a RS422/USB converter into a USB data packet. The Micro-Epsilon sensorTOOL V1.7.1 software recorded data at a sampling rate of 250 Hz.

### 3.3. Post-Process Line Distortion Measurement

**Figure 3:**
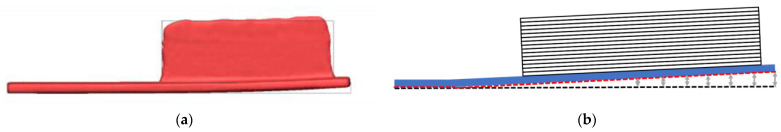
(**a**) Experimental optical 3D scanned data superimposed on reference CAD for Case 1; (**b**) Schematic showing the location for measurement of post-process line distortion.

After the deposition process was finished and the workpiece had cooled down, the workpiece was scanned with a Faro 3D Laser Scan Arm V3 Optical scanner, equipped with a scanning accuracy of 65 µm. Once the laser scanning had finished, Geomagic Control software as used to process the data obtained from the laser scanning. Then, the scanned data was compared with the workpiece CAD file that acted as a reference design in Geomagic Control. Then, the distribution was measured and analyzed experimentally at the line of the bottom surface of the substrate. The comparison of laser scanned data pertaining to the deposited wall and the workpiece’s CAD is shown in Figure 3a. Distortion was analyzed and measured at the center line of the bottom face of the substrate, as shown in Figure 3b.

## 4. Numerical Implementation

### 4.1. FEA Solver

The COMSOL Multiphysics-based solver (PARDISO) was employed to perform the FEM analysis. To reduce the computation time, the adaptive time stepping method was used, rather than a strict formulation. The computation time step of $R/v_{s}$ was taken during the material deposition period, and $3R/v_{s}$ during the dwell time period. This feature of adaptive time stepping should be noted when performing a comparison with experimental results in the coming section. All simulations were performed on a workstation equipped with 16 cores, 128 GB RAM, and an Intel Xeon W-2275 processor.

### 4.2. FEM Mesh

**Figure 4:**
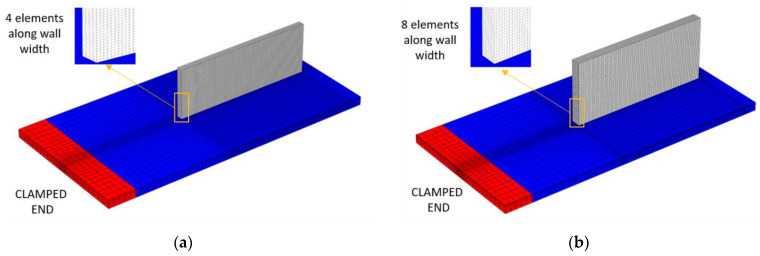
FE meshes used for the numerical simulation of LDED process (**a**) single-bead wall, (**b**) double-bead wall.

Figure 4 presents three-dimensional finite element meshes of both single- and double-bead walls, generated in COMSOL Multiphysics. The same mesh was used for the thermal model as well as the mechanical model. The mesh for a single-bead wall contained 48,370 Hex-8 elements and 60,480 nodes. Furthermore, the mesh for a double-bead wall contained 78,708 Hex-8 elements and 91,854 nodes. The mesh elements for the wall builds were taken as 2 per laser radius (0.525 mm and 0.425 mm for the single- and double-beads experiments, respectively), and 1 per layer thickness (0.428–0.561 mm, depending upon the experiment cases). The mesh element size in the deposited part (vertical wall) remained constant. However, a coarse mesh was used for the substrate, as the heat source moves along the center of the substrate, and hence requires a fine mesh size (high thermal gradient). Nevertheless, regions which are far away from the wall builds experience little of the thermal gradient; therefore, a coarse mesh was employed in those regions of substrate, thereby also contributing to a reduction in the computational cost.

### 4.3. Material Deposition Modeling

The deposition of material during the LDED process was simulated using the “quiet” element activation method [24]. The elements that represented the wall builds and substrate, as shown in Figure 4, were a part of the computation domain from the beginning of the analysis. However, dummy material properties (close to zero value of conductivity, specific heat, and Young’s Modulus) were assigned to the quiet elements so as to not affect the numerical results. For instance, the values of thermal conductivity k and specific heat C_P_ for quiet elements are rescaled as follows:

(12) $$k_{\mathrm{quiet}}={\;s}_{k}k$$

(13) $$C_{P}{}_{\mathrm{quiet}}={\;s}_{C_{P}}C_{P}$$

where $k_{\mathrm{quiet}}$ and $C_{P}{}_{\mathrm{quiet}}$ represent heat conductivity and specific heat, respectively, for the quiet elements; $s_{k}$ and $s_{C_{P}}$ are the scaling factors chosen, here equal to 10^−4^, as suggested in the literature [24], for both $s_{k}$ and $s_{C_{P}}$. Moreover, in the mechanical analysis, the material properties, such as stiffness or Young’s Modulus, are scaled in the same way. The change of material properties from “quiet” to “active” is complete when the following expression is satisfied (Equation (14)):

(14) $$\exp\left(-\frac{3{\left(x+v_{s}t\right)}^{2}}{a_{f,r}{}^{2}}-\frac{3y^{2}}{b^{2}}-\frac{3z^{2}}{c^{2}}\right)\;\geq 5\%$$

This means that if the DE heat source intensity at any node exceeds 5% of the peak intensity, as specified in the literature [24], the element is switched from the quiet to the active state. The existing quiet element is activated to become an active element in a state of zero stress; the temperature of quiet elements never exceeded 100 °C during the analysis due to the scaling factor $s_{k}$.

### 4.4. Model Calibrations and Boundary Conditions

To simplify the layer geometry, the deposited wall build is assumed to be parallelepipeds of constant height and width dimensions for all layers. However, this is contrary to the experimental trend, since the layer height is not uniform in the first few layers, as the process is not stable. As discussed in the previous sections, some input parameters need to be calibrated in order to develop an accurate thermo–mechanical model. For the DE heat source, a calibrated value of 0.4 is chosen for laser absorption efficiency (A) in order to obtain the best agreement between the calculated temperature and the experimental data recorded by thermocouple, as suggested in [5]. In the published literature employing the same process parameters, the authors have demonstrated the DE heat source parameters calibration techniques using experimentally measured melt-pool dimensions, obtained via thermal imaging camera and macrography [20]. The DE heat source dimensions parameters are chosen in such a way that they represent the same experimental melt-pool dimensions that lead to the front ellipsoid length $a_{f}={R\;\mathrm{and}\;a}_{r}=2a_{f},\;b=\;R\;\mathrm{and}\;c=1.3\times \mathrm{Layer}\;\mathrm{Height}$ for each experiment [20]. As is widely reported in the literature [25], a constant emissivity (ε) value of 0.6 is chosen. For the same reason, a constant convective heat transfer coefficient (h) is taken at the substrate, with h = 5 (W·m^−2^·K^−1^). At the wall builds, a greater value of convective heat transfer coefficient with h = 45 (W·m^−2^·K^−1^) is taken after reverse calibration, based on the literature that frequently reports values in the range of h = 20–60 (W·m^−2^·K^−1^) [6]. This large value aims to account for the effect of the powder-carrying gas that increases the heat losses around the melt pool surface. Heat losses are also present at the surfaces in contact with metallic fixtures clamps, as shown in Figure 2c. These losses are modeled with a formulation similar to the Newton’s law, using a value of 60 (W·m^−2^·K^−1^) for the convective heat transfer coefficient at the contact surfaces, as suggested in [7].

## 5. Results and Discussion

### 5.1. Thermal History

**Figure 5:**
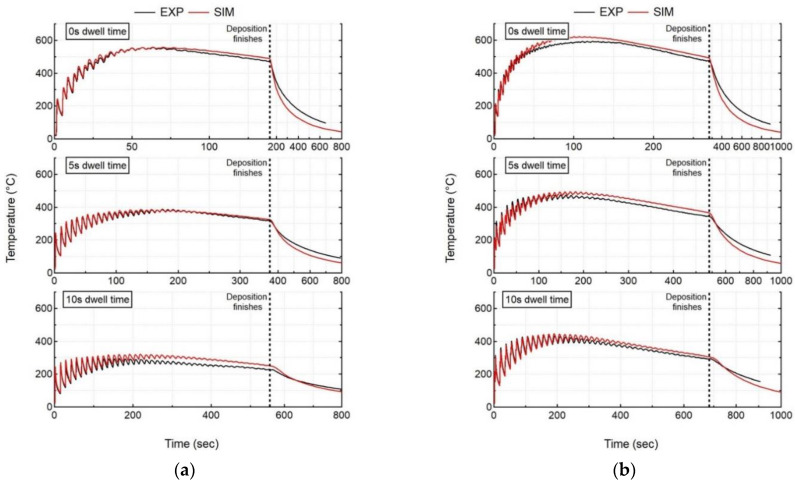
Comparison of numerical results (SIM) with recorded in situ thermal history (EXP) of thermocouple 1 for all cases (**a**) single-bead wall, (**b**) double-bead wall.

**Figure 6:**
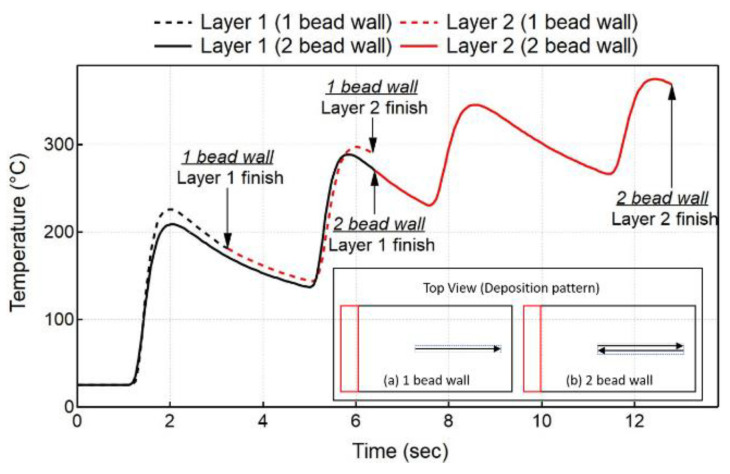
Comparison of temperature evolution between single- and double-bead wall at TC1 location during the deposition of the first two layers.

The thermal model predicts the thermal response of the workpiece, which is compared to the experimental measurements for all cases. Figure 5 shows the experimental results, as measured by thermocouple 1 (TC1), compared to the numerical results at the nodes corresponding to the thermocouple locations for all experiment cases. In order to highlight the deposition process, all of the graphs have a double scale X axis. As explained in the previous section (Experiment Set-up), indeed, thermocouples 1 and 2 are at different locations on the bottom face of the substrate; however, they are located along the deposition line. Hence, they record almost the same temperature evolution, but with a different time offset. Therefore, for this reason, only the thermocouple 1 results are presented in the graphs. The temperature analysis of the initial two layers is subsequently presented in Figure 6. The deposition pattern is also highlighted in Figure 6 (a,b).

A shorter dwell time (t_DW_) results in higher peak temperatures exceeding 550 °C (Case 1) and 600 °C (Case 4) for single- and double-bead walls, respectively. An increase in t_DW_ results in a lower peak temperature exceeding 250 °C (Case 3) and 450 °C (Case 6) for both single- and double-bead walls, respectively, since the already deposited material has more time to cool down before switching the laser on again. An increase in the number of beads in wall builds leads to higher deposited volume, which also plays a significant role in the thermal evolution in the workpiece. An increase in the number of beads results in higher peak temperatures for all t_DW_ cases. Single-bead walls experience peak temperatures of 550, 390, and 270 °C for 0, 5, and 10 s, respectively. However, double-bead walls experience higher peak temperatures of 600, 500, and 420 °C for 0, 5, and 10 s, respectively. For double-bead walls, there is no dwell time between the first and second bead deposition, thereby depositing the second bead beside the first bead without allowing it to cool down. The single wall also experiences the same phenomenon where layer 1 (1 bead) does not have the same cooling time as layer 2 (1 bead), which starts to deposit above layer 1 without allowing it to cool. However, double-bead walls deposit more volume as compared to a single-bead wall, leading to a different thermal evolution in the workpiece. This can be observed in Figure 6, which shows the comparison of the temperature evolution at the TC1 location during the deposition of the first two layers for single- and double-bead walls. The temperature obtained at the end of the second layer is much higher for the double-bead wall, as compared to a single-bead wall. This trend is also followed for the deposition of the complete wall.

The thermal results of the transient heat transfer analysis are in close agreement with the experimental results of the thermocouple presented in Figure 5. The average deviation between the experiment and simulation results are calculated by comparing the computation instances in time. This is why the experiment results are linearly re-sampled over time.

(15) $$\mathrm{Average}\;\mathrm{deviation}=\frac{\sum_{i=1}^{n}\left|{\left(T_{\exp}\right)}_{i}-{\left(T_{\mathrm{sim}}\right)}_{i}\right|}{n}$$

where n is the total number of simulation time increments between the start and end of the computation analysis that depends upon the dwell time, number of beads etc. (n range from 2151–4138 for Case 1–6), i is the current time increment, $T_{\exp}$ is the measured temperature, and $T_{\mathrm{sim}}$ is the simulated temperature. The largest average deviation at thermocouple 1 is found to be 13.2 °C for Case 3. Table 3 shows the computation time and average deviation at thermocouple 1 for all experiment cases.

### 5.2. Mechanical History

**Figure 7:**
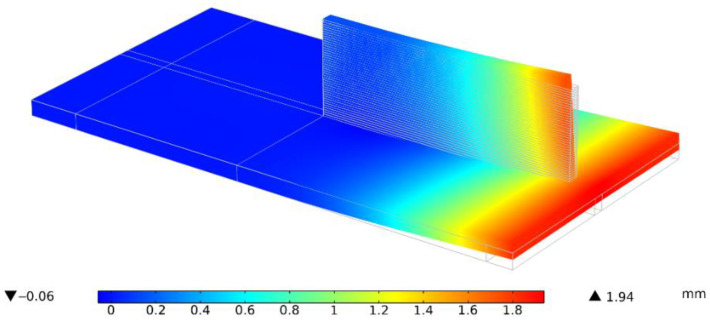
Mechanical model showing the final deformed configuration for Case 1.

The workpiece experiences repeated thermal expansion and shrinkage behavior due to the repeated thermal cycle of heating and cooling, respectively. This leads to the continuous accumulation of distortion throughout the process. Figure 7 shows the final calculated deformed configuration for a single-bead wall with no dwell time (Case 1).

#### 5.2.1. In Situ Distortion

**Figure 8:**
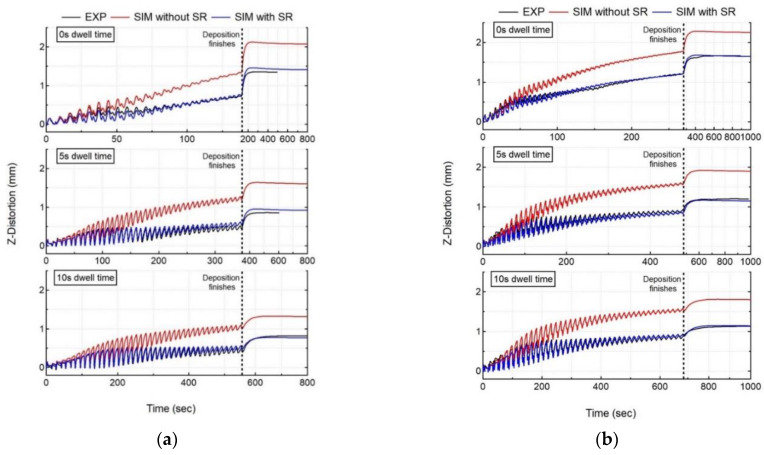
Comparison of numerical results (SIM with and without stress relaxation (SR)) with recorded in situ distortion accumulation of LDS (EXP) for all cases (**a**) single-bead wall, (**b**) double-bead wall.

The mechanical response of the workpiece is calculated by the mechanical model, and is then compared to the experimental measurements for all cases. Figure 8 shows the experimental results of in situ distortion at the free end of the substrate measured by LDS, compared to the numerical results at the node corresponding to the LDS location for all experiment cases.

The laser and powder are co-focused on the substrate’s top face, and their interaction leads to material fusion with a high melt-pool temperature. Due to the high thermal diffusivity of SS 316L, the bottom face of the substrate also experiences an increase in temperature, as shown in Figure 5, that presents thermal evolution at the thermocouple location, but much lower than the fusion temperature. This causes a larger thermal expansion at the top surface relative to the bottom surface of the substrate, causing the substrate to deflect downwards. The downward deflection of substrate is recorded by LDS as a decrease in distortion values, as shown in Figure 8. Once the layer deposition is finished, the melt-pool and substrate begin to cool, leading to contraction, causing the free end of the substrate to deflect upwards. The upward deflection of substrate is recorded by LDS as an increase in distortion values, as shown in Figure 8.

For all experiment cases, independent of dwell time and the number of beads, it is observed that the distortion trend and accumulation are consistent throughout the deposition process. However, the distortion magnitude accounting for each layer starts to decrease after the deposition of 20–22 layers, as the heat source keeps moving up from the substrate. For SS 316L with cantilever tooling, an increase in dwell time results in a decrease of distortion for both single- and double-bead walls. However, increasing the number of beads results in an increase of distortion values for all dwell time cases. Figure 8 shows the numerical distortion results (SIM) both with and without stress relaxation (SR) compared with experimental results (EXP). Table 4 shows the computation time, average deviation (averaged over all LDS measured values), and final error (last measurement recorded by LDS) at the LDS location for all experiment cases. Both models correctly capture the distortion trend throughout the deposition correctly. However, the numerical model without stress relaxation does not capture the distortion magnitude correctly, as it predicts significantly higher levels of distortion, as compared with experiment data for all cases (error > 50%). It captures the distortion magnitude correctly for the first few layers, but then starts to overpredict for the subsequent layer’s deposition, suggesting that the effect of annealing and liquefaction induced stress relaxation in LDED. Unlike the numerical model without stress relaxation, the numerical model with stress relaxation correctly captures both the distortion trend and magnitude for the complete deposition process (error < 10%). This justifies the need for including the effect of liquefaction and annealing-induced stress relaxation in the workpiece. The numerical model with stress relaxation captures the thermal expansion and shrinkage trends correctly, in close agreement with the experiment results for all cases.

#### 5.2.2. Post-Process Line Distortion

The mechanical model’s calculated distortions (Figure 7) on a part scale show good agreement with the experiment post-process distortions measured by the optical scanner, as shown in Figure 3a, for all experiment cases.

**Figure 9:**
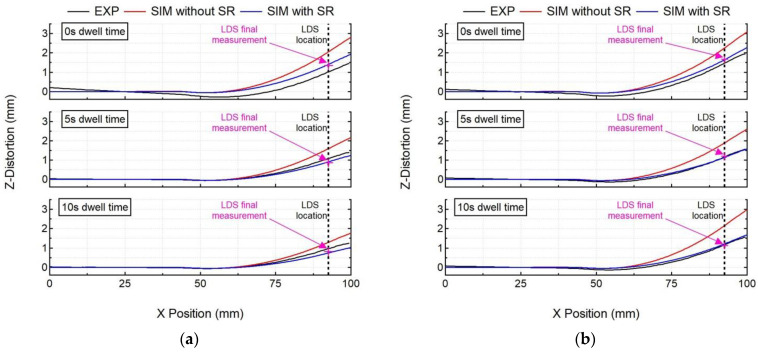
Comparison of numerical results (SIM with and without stress relaxation (SR)) with experimental post-process line distortion results of the optical scanner (EXP) for all cases (**a**) single-bead wall, (**b**) double-bead wall.

The numerical model with stress relaxation correctly captures the post-process distortion shape and magnitude for all cases, as shown in Figure 9 (error < 15%), with the exception of experiment Case 1 (error: 32%). One of the possible reasons for this discrepancy can be attributed to the release of residual stresses during unclamping that contributes to distortion. Perhaps this effect is not captured by the model accurately, leading to error. However, globally, the numerical model with stress relaxation performs well with a maximum error of 15% in cases 2–4. For Cases 5–6, computation error is less than 5%. The numerical model without stress relaxation significantly over-predicts the distortion magnitude for all cases (error 40%). The computation error ranges from 42% (Case 3) to 93% (Case 1), thereby justifying the need to include stress relaxation in the numerical model. As was observed with the in situ distortion, post-process distortion also follows the same trend—an increase in dwell time results in a decrease in post-process distortion for both single- and double-bead walls. Furthermore, an increase in the number of beads results in an increase in post-process distortion values for all dwell time cases. These trends are well captured by the numerical model, and thereby shows its versatility. The final measurement recorded by the LDS sensor before the workpiece was unclamped is marked on Figure 9. This comparison highlights the fact that the LDS sensor and the optical 3D scanner provide consistent values of distortion.

## 6. Simulation Speed-Up

Due to the feature of the big track size in LDED, combined with the specified computational time increment of $R/v_{s}$, this leads to a high computation time, as given in Table 3 and Table 4. Therefore, to reduce the computation time, an elongated ellipsoid (EE) line heat input model is employed that averages the heat source over its deposition path [26]:

(16) $$Q_{\mathrm{EE}}=\frac{6\sqrt{3}\mathrm{AP}}{\hat{a}\mathrm{bc}\pi \sqrt{\pi }}\exp\left(-\frac{3{\left(x+{\;v}_{s}\left(t\;+\frac{1}{2}\Delta t\right)\right)}^{2}}{{\hat{a}}^{2}}-\frac{3y^{2}}{b^{2}}-\frac{3z^{2}}{c^{2}}\right)$$

where the length of each EE sub-track or segment $\hat{a}$ is [26]:

(17) $$\hat{a}=\frac{v_{s}\Delta t}{2}\sqrt{\frac{3}{\log2}}$$

Instead of taking hundreds of computational time steps employing the DE heat source, with the EE model, large computational time steps are possible in the analysis. However, employing such large computational time increments also leads to large computation average deviation [26]. Therefore, it is recommended to divide the deposition scan (track length) into multiple successive linear scans (sub-tracks) along the deposition path. For each individual linear scan, the EE source is applied in one computational time increment. Hence, different track sizes (sub-track) are chosen and investigated for the EE source, as suggested in the literature [20]. Material activation from the quiet to active state is completed with the same procedure as described in Section 4.3. Finally, a dimensionless number K_E_ is introduced [20] in order to compare different elongated lengths (sub-track).

(18) $$K_{E}=\frac{v_{s}\Delta t}{a}$$

Goldak’s double ellipsoid, or any other heat source model, requires $K_{E}\leq 1$ to simulate the continuous motion of the heat source without skipping over some elements. Table 5 presents different elongated lengths and parameters used in the present work.

**Figure 10:**
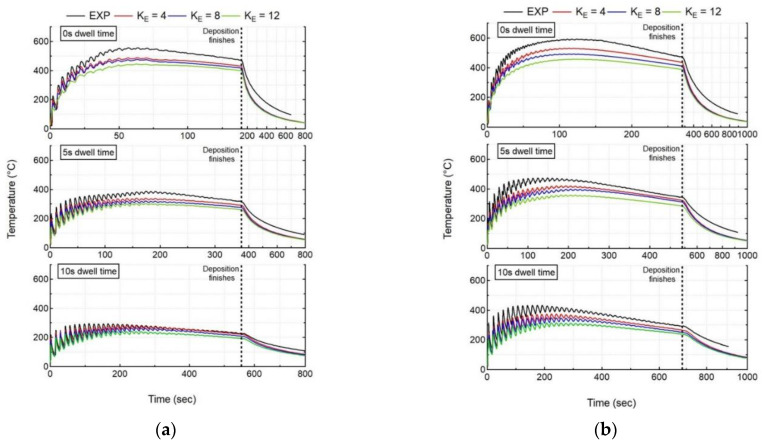
Comparison of numerical results using the elongated ellipsoid (EE) with recorded in situ thermal history of thermocouple 1 for all cases (**a**) single-bead wall, (**b**) double-bead wall.

With an increase of K_E_, the length of elongated ellipsoid ($\hat{a}$) increases lead to a reduction in computational time steps. However, this also leads to an averaging of laser energy over larger domains, thereby reducing the peak intensity of the elongated ellipsoid heat source, leading to an increase in computation average deviation. For all cases with different K_E_ values, the numerical model can capture the trends of temperature evolution; however, the mean and peak temperature values have been reduced to be proportional to K_E_, as shown in Figure 10.

**Figure 11:**
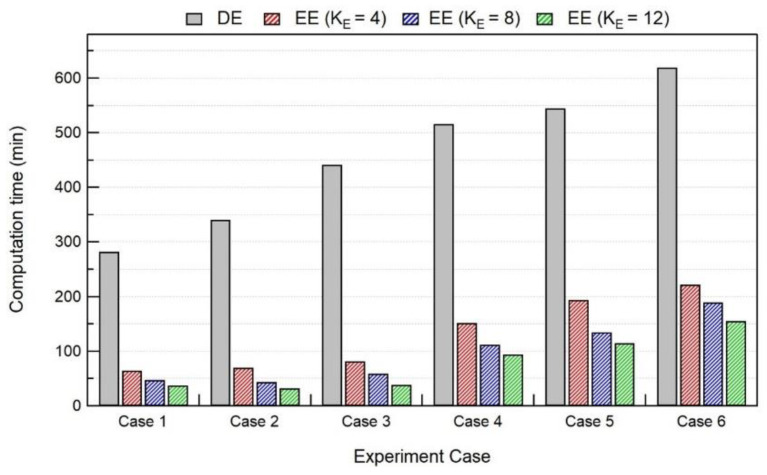
Comparison of computation time for thermal analysis between DE and EE heat sources with different elongated length.

It can be noted that, with an increase in K_E_, the computation average thermal deviation at the thermocouple location (global level) starts to increase, as shown in Figure 10. This trend of increasing computation average thermal deviation with an increase in K_E_ also happens at the melt-pool level. However, with the introduction of K_E_, the computation time is drastically reduced up to a factor of 5–10, as presented in Figure 11. A comparison of computation time for thermal analysis is performed between the double ellipsoid (DE) and elongated ellipsoid (EE) with different K_E_ values. With an increase in K_E_, the computation time starts to decrease for all experiment cases. Considering all experiment cases, K_E_ = 4, 8 appears to give better thermal results, with a maximum average thermal deviation of 100 °C with K_E_ = 8. For all experiment cases, the average deviation for in situ temperature results at the thermocouple location was less than 100 °C, using the elongated ellipsoid for K_E_ = 4, 8 values.

**Figure 12:**
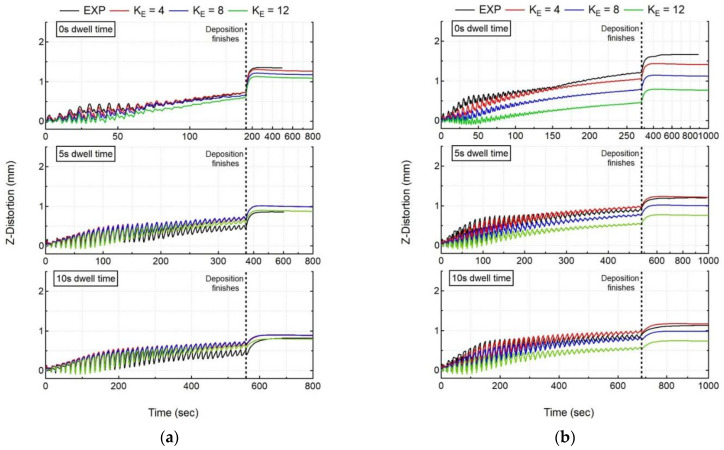
Comparison of numerical results using the elongated ellipsoid with recorded in situ distortion accumulation of LDS for all cases (**a**) single-bead wall, (**b**) double-bead wall.

For the mechanical analysis, as explained in the previous section, the layer deposition/fusion leads to a lowering of the substrate. This movement is recorded as a decrease in the distortion values, as explained in the previous section. Due to the elongated ellipsoid length (K_E_), the peak temperatures obtained during the layer deposition are significantly reduced. This leads to a reduction in the phenomenon of the lowering of the substrate, accounting for a decrease in distortion. Moreover, the cooling phenomenon is altered due to the lower temperature field obtained with different K_E_ values. For all cases, as shown in Figure 12, with different K_E_ values, the numerical model can capture the in situ distortion evolution; however, the magnitude of the distortion values changes with respect to K_E_. Considering all experiment cases, K_E_ = 4, 8 appears to provide acceptable distortion results (errors < 15%), with an exception in Case 4 (error: 33.5%), resulting in a maximum average distortion deviation of 0.56 mm with K_E_ = 8. For all other cases, the average deviation for in situ distortion is less than 0.25 mm (errors < 15%) using an elongated ellipsoid for K_E_ = 4, 8 values.

**Figure 13:**
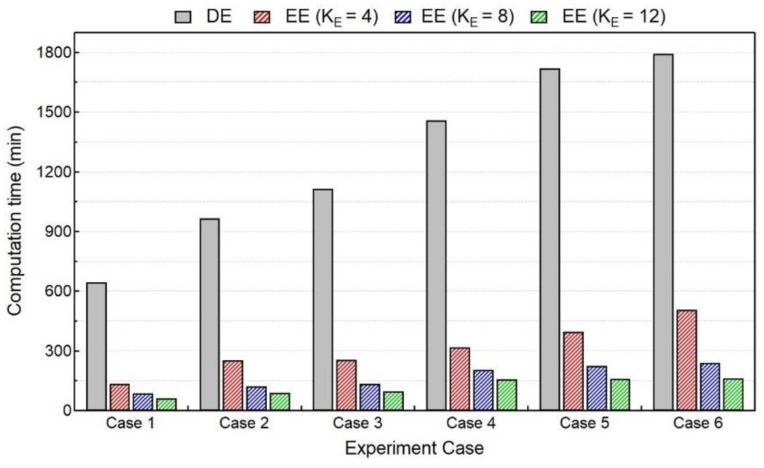
Comparison of computation time for mechanical analysis between DE and EE heat sources with different elongated length.

However, with the introduction of the elongated ellipsoid, the reduction in computation time follows the same trend as thermal analysis. As shown in Figure 13, for the mechanical analysis, the computation time is reduced up to a factor of 5–10 with K_E_ = 4, 8 values.

**Figure 14:**
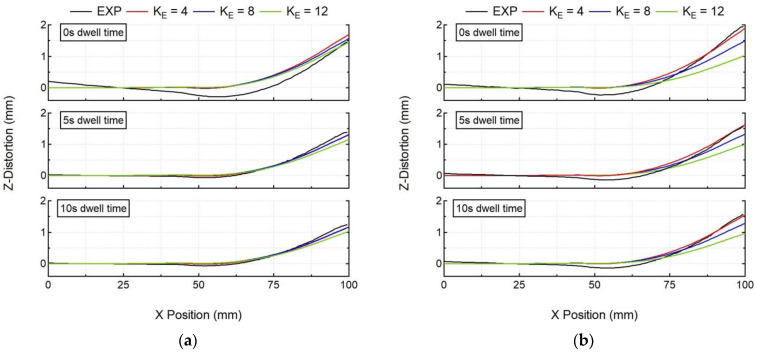
Comparison of numerical results using the elongated ellipsoid with experimental post-process line distortion results of the optical scanner for all cases (**a**) single-bead wall, (**b**) double-bead wall.

The above-mentioned numerical results (Figure 12) for in situ distortion obtained via the elongated ellipsoid heat source show acceptable levels of accuracy, but with drastically reduced computation times (Figure 13). These results are also verified with as post-process line distortion results, as shown in Figure 14. Here, the numerical model with K_E_ = 4.8 values, for all experiment cases, also predicts the substrate line deformation, which is in good agreement with the experiment results. Therefore, a thermo–mechanical model with a calibrated elongated ellipsoid heat source can be helpful in predicting the workpiece deformation in a practical computation time.

## 7. Conclusions

In the present work, a 3D thermo-elasto-plastic FE model is developed to analyze the in situ and post-process distortion accumulated in the LDED process. The effect of inter-layer dwell time and the number of beads is studied. The thermo–mechanical model is validated for different inter-layer dwell times and the number of beads. The main conclusions of this work are the following:

1. The computed temperature history predicted by the thermal model is in good agreement. The maximum average deviation at the thermocouple location is 13.2 °C, in comparison with the experiment measurements (Case 3).
2. The mechanical model with stress relaxation is in good agreement with in situ and post-process distortion measurements. The maximum average deviation of in situ distortion at the LDS location without the stress–relaxation model is 0.313 mm, while with the stress–relaxation model, it is 0.041 mm, in comparison with the experiment measurements (Case 6), with the computation average deviation reduced to a factor of 8. The model without SR over-predicted the distortion by 35–85%, and the model with SR yielded much higher computational accuracy (maximum error of 9.4% in Case 3).
3. The computed distortion without stress relaxation is significantly over-estimated, as it does not include the effects of liquefaction and process-induced annealing behavior in LDED. However, by using the stress–relaxation model, the computed distortion is in good agreement with the experiment results.
4. For the cantilever tooling with the SS 316L material, with an increase in the inter-layer dwell time, distortion decreases, and with an increase in the number of beads, distortion increases. The numerical model demonstrated its versatility by capturing these trends with good accuracy.
5. The computation time can be reduced drastically by a factor of 10 using the EE heat source model. Without considering the exception (Case 4 with K_E_ = 8), the EE model with K_E_ = 4 and 8 values results in a maximum average deviation of 0.25 mm. The EE model with K_E_ = 4 and 8 values yields computation errors (LDS) of less than 15% (with the exception of Case 4). The local accuracy of the model (temperature, distortion) may be affected, but the global values of temperature and distortion are in agreement with the experiment measurements.
6. Large-part simulation can be performed with a reasonable computation time when the EE heat source model is employed.

## Figures and Tables

**Table 1:** Temperature-dependent material properties of stainless steel 316L [11].

T (°C)	k (W·m^−1^·K^−1^)	C_p_ (J·kg^−1^·K^−1^)	E (GPa)	α (10^−5^·K^−1^)	$\sigma_{Y}$ (MPa)
20	14	450	192	1.4	275
100	15.1	490	186	1.5	238
200	16.4	522	178	1.6	198
300	17.8	545	170	1.7	172
400	19.1	555	161	1.7	157
500	20.5	566	153	1.8	151
600	21.8	583	145	1.8	145
700	23.2	600	137	1.8	136
800	24	614	110	1.9	127
900	25.9	629	63	1.9	115
1000	27	643	37	1.9	78
1100	28.6	657	16	1.9	38
1200	29.9	671	11	2	24
1300	31.3	686	8	1.8	20
1400	32.6	700	8	1.8	16

**Table 2:** Description of the experiment cases with process parameters.

Case	No. of Beads	Dwell Time (s)	Wall Length (mm)	Wall Width (mm)	Wall Height (mm)
1	1	0	50	2.1	18
2	1	5	50	2.1	18.1
3	1	10	50	2.1	18.2
4	2	0	50	3.4	23.2
5	2	5	50	3.4	23.5
6	2	10	50	3.4	23.6

**Table 3:** Experiment cases examined for thermal model validation via thermocouple.

Case	No. of Beads	Dwell Time (s)	Computation Time	Average Deviation (°C)
1	1	0	4 h 42 min	4.2
2	1	5	5 h 41 min	5.2
3	1	10	7 h 22 min	13.2
4	2	0	8 h 36 min	5
5	2	5	9 h 5 min	6.4
6	2	10	10 h 20 min	6.6

**Table 4:** Experiment cases examined for mechanical model validation via LDS.

Case	No. of Beads	Dwell Time (s)	Computation Time	Average Deviation (mm)		Error (%)	
				No SR	With SR	No SR	With SR
1	1	0	10 h 45 min	0.185	0.029	52.9	3.9
2	1	5	16 h 7 min	0.25	0.06	85.3	4.9
3	1	10	18 h 37 min	0.17	0.042	57.8	9.4
4	2	0	24 h 19 min	0.12	0.069	34.7	1.8
5	2	5	28 h 41 min	0.27	0.049	57.5	5.8
6	2	10	29 h 55 min	0.313	0.041	58.9	0.3

**Table 5:** Elongated ellipsoid (EE) heat source model parameters used in the present work.

K_E_	Computation Time Step FEM (∆t) (s)	EE Length ($\hat{a}$) (mm)	No. of Sub-Tracks per Layer (Wall Length/$\hat{a}$)	
			1 Bead Wall	2 Bead Wall
4	0.528	9.15	6	12
8	1.056	18.3	3	6
12	1.584	27.46	2	4

## Data Availability

Not applicable.
